# Cocaine-Induced Changes in Tonic Dopamine Concentrations Measured Using Multiple-Cyclic Square Wave Voltammetry *in vivo*

**DOI:** 10.3389/fphar.2021.705254

**Published:** 2021-07-06

**Authors:** Jason Yuen, Abhinav Goyal, Aaron E. Rusheen, Abbas Z. Kouzani, Michael Berk, Jee Hyun Kim, Susannah J. Tye, Charles D. Blaha, Kevin E. Bennet, Dong-Pyo Jang, Kendall H. Lee, Hojin Shin, Yoonbae Oh

**Affiliations:** ^1^ Department of Neurologic Surgery, Mayo Clinic, Rochester, MN, United States; ^2^ Deakin University, IMPACT—the Institute for Mental and Physical Health and Clinical Translation, School of Medicine, Barwon Health, Geelong, VIC, Australia; ^3^ Medical Scientist Training Program, Mayo Clinic, Rochester, MN, United States; ^4^ School of Engineering, Deakin University, Geelong, VIC, Australia; ^5^ Queensland Brain Institute, The University of Queensland, St Lucia, QLD, Australia; ^6^ Division of Engineering, Mayo Clinic, Rochester, MN, United States; ^7^ Department of Biomedical Engineering, Hanyang University, Seoul, Korea; ^8^ Department of Biomedical Engineering, Mayo Clinic, Rochester, MN, United States

**Keywords:** cocaine, tonic dopamine, addiction, voltammetry, nucleus accumbens, neuroscience, psychiatry, mental disorders

## Abstract

For over 40 years, *in vivo* microdialysis techniques have been at the forefront in measuring the effects of illicit substances on brain tonic extracellular levels of dopamine that underlie many aspects of drug addiction. However, the size of microdialysis probes and sampling rate may limit this technique’s ability to provide an accurate assessment of drug effects in microneural environments. A novel electrochemical method known as multiple-cyclic square wave voltammetry (M-CSWV), was recently developed to measure second-to-second changes in tonic dopamine levels at microelectrodes, providing spatiotemporal resolution superior to microdialysis. Here, we utilized M-CSWV and fast-scan cyclic voltammetry (FSCV) to measure changes in tonic or phasic dopamine release in the nucleus accumbens core (NAcc) after acute cocaine administration. Carbon-fiber microelectrodes (CFM) and stimulating electrodes were implanted into the NAcc and medial forebrain bundle (MFB) of urethane anesthetized (1.5 g/kg i.p.) Sprague-Dawley rats, respectively. Using FSCV, depths of each electrode were optimized by determining maximal MFB electrical stimulation-evoked phasic dopamine release. Changes in phasic responses were measured after a single dose of intravenous saline or cocaine hydrochloride (3 mg/kg; *n* = 4). In a separate group, changes in tonic dopamine levels were measured using M-CSWV after intravenous saline and after cocaine hydrochloride (3 mg/kg; *n* = 5). Both the phasic and tonic dopamine responses in the NAcc were augmented by the injection of cocaine compared to saline control. The phasic and tonic levels changed by approximately x2.4 and x1.9, respectively. These increases were largely consistent with previous studies using FSCV and microdialysis. However, the minimal disruption/disturbance of neuronal tissue by the CFM may explain why the baseline tonic dopamine values (134 ± 32 nM) measured by M-CSWV were found to be 10-fold higher when compared to conventional microdialysis. In this study, we demonstrated phasic dopamine dynamics in the NAcc with acute cocaine administration. M-CSWV was able to record rapid changes in tonic levels of dopamine, which cannot be achieved with other current voltammetric techniques. Taken together, M-CSWV has the potential to provide an unprecedented level of physiologic insight into dopamine signaling, both *in vitro* and *in vivo*, which will significantly enhance our understanding of neurochemical mechanisms underlying psychiatric conditions.

## Introduction

Substance dependence is a global public health problem. A 2019 survey revealed 20.4 million people aged 12 or older in the United States suffered from substance use disorder (Substance Abuse and Mental Health Services Administration, 2020). Around 40–60% of patients experience relapse within one year of treatment discharge (McLellan et al., 2000), which is hypothesized to be a result of long-term neuroplastic changes after chronic drug use (Kalivas and O'Brien, 2008). Therefore, it is important to understand the neurobiology of addiction in order to improve our treatment strategies.

Dopamine is widely implicated in addiction. Its functions include determining the incentive value of naturally occurring positive rewarding stimuli (e.g., food, water, and conspecific mates) (Blaha and Phillips, 1996). Previous studies have also demonstrated that dopamine release in the nucleus accumbens, dorsal striatum, and the prefrontal cortex is a cardinal feature in models of addiction, with dopamine receptor blockade in these areas disrupting drug-seeking behaviors (Berke and Hyman, 2000; Ito et al., 2002; Vanderschuren et al., 2005; Murray et al., 2012; Zbukvic et al., 2016; Hodebourg et al., 2019). However, measuring dopamine with high temporal and spatial resolution *in vivo* is a major challenge.

There are generally two distinct patterns of spike firing exhibited by neuronal dopamine-containing cells in the mammalian midbrain: tonic activity and phasic burst activity (Grace, 1991; Grace, 2016). Phasic activity causes a transient and robust release of dopamine in the synapse that serves as a learning signal for neural plasticity (Schultz, 2007). Tonic activity refers to continuous spontaneous extra-synaptic dopamine release driven by pacemaker-like firing of dopamine neurons, providing a relatively homeostatic extracellular level of dopamine (i.e., tonic concentration) in the striatum thought to modulate behavioral flexibility (Goto et al., 2007).

Tonic concentrations of dopamine in the brain have been typically quantified in the sub-nM range using microdialysis (Watson et al., 2006; Gu et al., 2015). Microdialysis has been utilized for sampling neurochemical substances, such as dopamine with high selectivity and sensitivity. However, it has several drawbacks when compared to electrochemical techniques (Robinson et al., 2003; Heien et al., 2004; Chefer et al., 2009; Rodeberg et al., 2017; Kim et al., 2021). These include limited temporal resolution ( ≥ 1 min) in comparison to voltammetry (milliseconds to seconds), and the relatively large dimensions of dialysis probes (typically > 200 μm), resulting in variable physicochemical characteristics, tissue damage, and relatively low spatiotemporal resolution (Morelli et al., 1992; Di Chiara et al., 1993; Blaha et al., 1996; Chefer et al., 2009; Oh et al., 2018; Rusheen et al., 2020). For these reasons, and the fact that it requires continuous sampling from the brain and laboratory analysis, its application in the human nervous system is limited.

In contrast, electrochemical methods, such as fast-scan cyclic voltammetry (FSCV), have features that are well-suited to quantitatively measure changes in extracellular dopamine concentrations (Millar, 1997; Robinson et al., 2003; Huffman and Venton, 2009; Lama et al., 2012). FSCV provides excellent temporal resolution (milliseconds time response) and detection sensitivity ( <5 nM). In this technique, a carbon-fiber microelectrode (CFM, diameter typically <10 μm) is held at a resting potential and then ramped to an electric potential sufficient to oxidize and reduce the electroactive species before the potential is returned to the resting potential (Robinson et al., 2003). This results in a measured current, which yields a cyclic voltammogram. The electrical scan takes less than 10 ms and is repeated every 100 ms (corresponding to a rate of 10 Hz). The voltammogram gives a chemical signature, which can be used to identify the chemical species of interest and quantify phasic changes in extracellular concentration. However, because of a large capacitive current which must be subtracted out to resolve the faradaic current, the application of conventional FSCV provides only measurements of phasic changes in neurochemical concentrations (Howell et al., 1986). FSCV cannot measure dysregulation in tonic concentrations of neurotransmitters (minutes to hours) that are likely to be important characteristics of many neurologic and psychiatric conditions (Dreher and Burnod, 2002; Berke, 2018).

For the measurement of tonic dopamine levels in the brain in real time, several electrochemical techniques were developed such as fast-scan controlled-adsorption voltammetry (FSCAV) (Atcherley et al., 2013), square wave voltammetry (Taylor et al., 2019), and convolution-based current removal technique (Johnson et al., 2017). Among these techniques, FSCAV from Heien and colleagues has been applied to study tonic dopamine levels in various experiment setups (Atcherley et al., 2015; Burrell et al., 2015; Abdalla et al., 2017). FSCAV utilizes adsorption properties of dopamine to the carbon microelectrode using multiple conventional FSCV waveforms. We have previously developed a technique that uses cyclic square wave voltammetric waveforms, called Multiple-Cyclic Square Wave Voltammetry (M-CSWV). The time resolution is 10 s, which is slower than FSCV but is well-matched to the timescale of changes in tonic concentrations of dopamine relevant to the pathologies of interest (Schultz, 2007). Since M-CSWV utilizes square waveforms, M-CSWV is able to harvest higher dimensional data for analysis that leads to higher sensitivity and selectivity than other tonic level measurement techniques (Kim et al., 2019; Kim et al., 2021).

In this study, we aim to elucidate the acute effects of cocaine administration on phasic dopamine release by using FSCV and tonic dopamine levels by using M-CSWV in the nucleus accumbens core (NAcc). Cocaine is one of the most common illicit drugs with an increasing prevalence of use and dependence (John and Wu, 2017).

## Materials and Methods

### Animal Subjects

Nine male Sprague-Dawley rats (Envigo, United States) were used for this study. Rats were kept in social housing in an Association for Assessment and Accreditation of Laboratory Animal Care International (AAALAC) accredited vivarium following a standard 12-h light/dark cycle at constant temperature (21°C) and humidity (45%) with ad libitum food and water. The present studies were approved by the Institutional Animal Care and Use Committee (IACUC), Mayo Clinic, Rochester, MN. The NIH Guide for the Care and Use of Laboratory Animals guidelines (Department of Health and Human Services, NIH publication No. 86-23, revised 1985) were followed for all aspects of animal care.

### Electrode Fabrication

CFMs were fabricated using an established standardized CFM design at Mayo Clinic. (Chang et al., 2013; Oh et al., 2016). Briefly, each microelectrode involved isolating and inserting a single carbon fiber (AS4, diameter = 7 μm; Hexel, Dublin, CA) into a silica tube (20 µM ID, 90 µM OD, 10 µM coat with polyimide; Polymicro Technologies, Phoenix, AZ). The connection between the carbon fiber and the silica tubing was covered with epoxy resin. The silica tubing was then attached to a nitinol (Nitinol #1, an alloy of nickel and titanium; Fort Wayne Metals, IN) extension wire with a silver-based conductive paste (Chang et al., 2013). The carbon fiber attached nitinol wire was insulated with polyimide tubing (0.0089″ID, 0.0134″OD, 0.00225″ WT; Vention Medical, Salem, NH) up to the carbon fiber sensing part. The exposed carbon fiber was then trimmed under a dissecting microscope to a length of 50 µm. Teflon-coated silver (Ag) wire (A-M systems, Inc., Sequim, WA) was prepared as an Ag/AgCl counter-reference electrode by chlorinating the exposed tip in saline with a 9 V dry cell battery. CFMs were pretested in a flow cell prior to coating deposition with a PEDOT:Nafion deposition solution (Vreeland et al., 2015), which minimized the effect of *in vivo* biofouling.

### Implantation of Recording and Stimulating Electrodes

**FIGURE 1:**
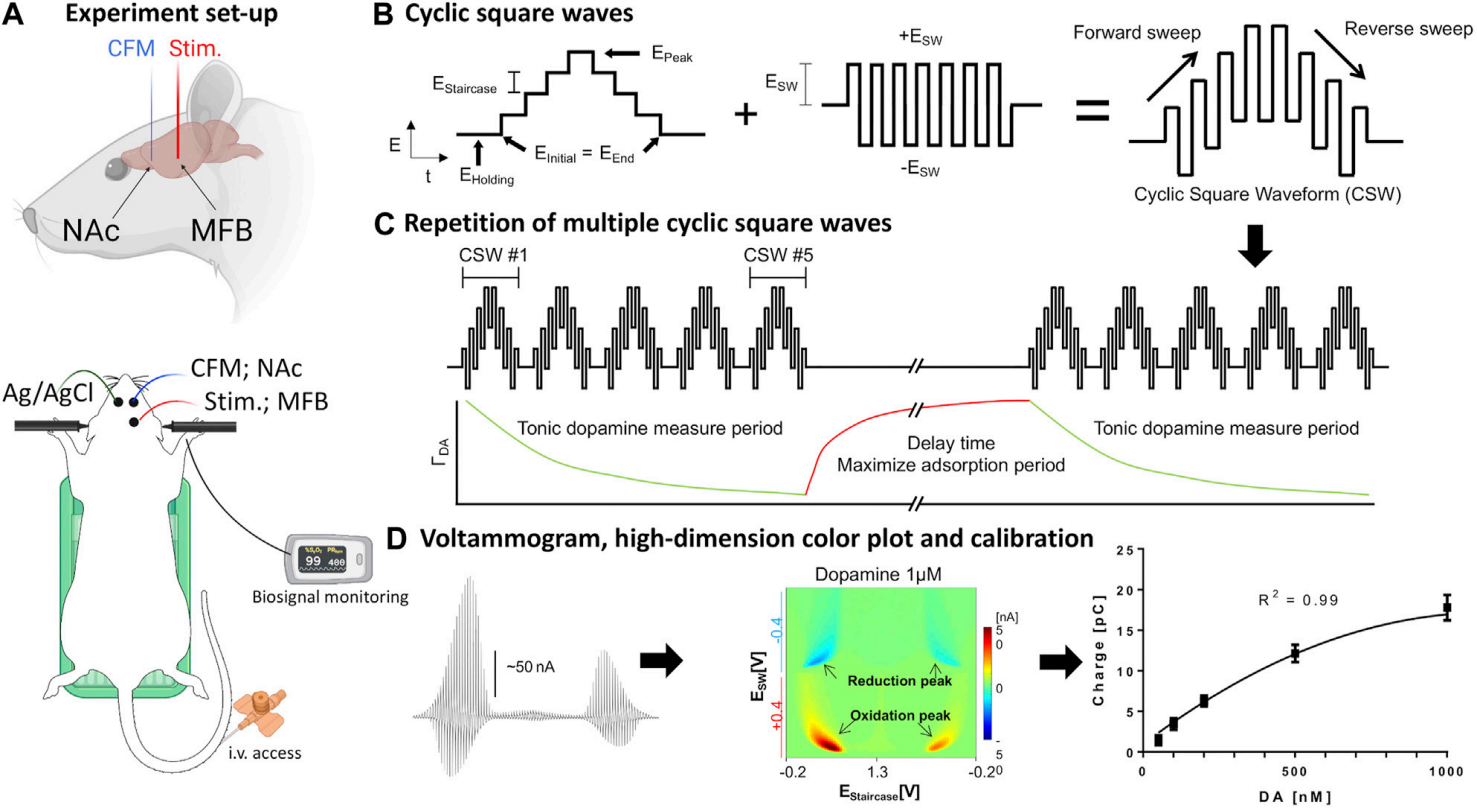
Set-up of *in vivo* voltammetry experiment. **(A)** Rat surgery set-up. Recording and stimulating electrodes were inserted unilaterally into the core of the nucleus accumbens and medial forebrain bundle, respectively. The counter-reference Ag/AgCl electrode was inserted contralaterally into cortical tissue. The rat is placed in a stereotactic frame with tail vein access, heating pad, and pulse oximetry monitoring. **(B,C)** Schematic design of waveform-CSWV applied to the CFM and its response. **(D)** Left-to-right: Raw voltammogram after removal of background currents, high-dimensional pseudo-color plot, M-CSWV signal calibration with tonic dopamine experiment (*n* = 4 electrodes). Figures adopted from (Oh et al., 2018) with permission. *Ag/AgCl*, silver chloride reference electrode; *CFM*, carbon-fiber electrode; *CSW*, cyclic square wave; *MFB*, medial forebrain bundle; *NAc*, nucleus accumbens; *stim.*, bipolar stimulating electrode. Parts of the Figure were created with Biorender.com.

Each rat was anesthetized with urethane (1.5 g/kg i.p.; Sigma-Aldrich, St Louis, MO) and administered buprenorphine (0.05–0.1 mg/kg s.c, Par Pharmaceutical, Chestnut Ridge, NY, United States) for analgesia. Following anesthesia, they were placed in a stereotaxic frame (David Kopf Instruments, Tujunga, CA). Respiratory rate (RespiRAT, Intuitive Measurement Systems), hind-paw and tail pinch were used to monitor the physiological state and depth of anesthesia. Using a standard rat atlas (Paxinos and Watson, 2007), three trephine holes were drilled, the first for placement of a CFM into the NAcc (AP 1.2 mm, ML 2.0 mm, DV 6–7 mm from dura), the second for a stimulating electrode into the medial forebrain bundle (MFB) (twisted bipolar stimulating electrode—Plastics One, MS 303/2, Roanoke, VA, with the tips separated by 1 mm; AP −4.6 mm, ML 1.3 mm, DV 8–9 mm from dura), and a third for an Ag/AgCl into the contralateral cortex (Clark et al., 2010) (Figure 1A).

### Drug Administration and Recordings

The stimulating electrode in the MFB and CFM in the NAcc were first adjusted to obtain a robust stimulation-evoked dopamine signal via FSCV (−0.4–1.3 V sweep; 10 Hz). MFB was chosen as it is known to induce dopamine release in the NAcc (Ng et al., 1991; Shu et al., 2013). For the phasic dopamine group, evoked responses (60 Hz, 0.2 mA, 2 ms pulse width, 2 s duration) were recorded at pre-, 5, 10, and 20, 30 40, 50, 60 min post-injection. This was performed using WINCS Harmoni system (Lee et al., 2017), a wireless neurochemical sensing system. Cocaine hydrochloride (Sigma-Aldrich, St. Louis, MO) at a single bolus of 3 mg/kg i.v., was used. Cocaine hydrochloride was given for 5-min duration in all cases.

For the tonic dopamine group, the system was switched to the M-CSWV sensing technique with electrodes at the same position after identification of an optimal CFM position in the NAcc using FSCV. Cocaine at the same dose and route as above was administered after baseline and post-saline recording. Further recordings were performed for another 90 min to monitor the potentially lasting effects of cocaine on tonic dopamine levels. Dynamic background subtraction and capacitive background current modeling was used to eliminate large capacitive background currents, allowing tonic dopamine concentrations to be measured every 10 s (Oh et al., 2018). Because of the uniqueness of the waveform, the voltammetric outcome of M-CSWV provides a wealth of electrochemical information beyond that provided by conventional FSCV (Figures 1B–D).

### Calibration of Electrodes

After experimentation, changes in dopamine release for phasic studies were determined by calibration of CFMs using a flow cell injection apparatus; whereas for tonic dopamine levels, calibration with dopamine solutions were used (Oh et al., 2018). The media used consisted of TRIS buffer (15 mM tris, 3.25 mM KCl, 140 mM NaCl, 1.2 mM CaCl2, 1.25 mM NaH2PO4, 1.2 mM MgCl2, and 2.0 mM Na2SO4, with the pH adjusted to 7.4) (Oh et al., 2018).

### Modelling Dopamine Dynamics

Measurement of phasic dopamine release using FSCV offers many important applications, including modeling dopamine release and reuptake kinetics, and modeling the effects of pharmacologic agents on these processes. To quantitatively characterize the effects of cocaine administration on synaptic dopamine release, we used the restricted diffusion model of Walters et al. (Walters et al., 2015). This model proposes that the synapse-electrode system consists of two anatomically separated compartments and allows for restricted diffusion from the synaptic compartment to the electrode compartment. This allows for more accurate fitting of *in vivo* data compared to previous models such as the diffusion gap model. We applied the restricted diffusion model to our data to extract best-fit estimates for the parameters R_p_, k_R_, k_U_, and k_T_ (see Table 1 for more information). Best fit was evaluated with root mean square error (RMSE). The model was fit to five sets of stimulation-induced phasic dopamine releases with saline onboard, and five sets with cocaine on board, 5 min after administration.

**TABLE 1:** Parameters calculated for the FSCV response pre-cocaine and 10 min post-cocaine, based on the model by Walters et al. (Walters et al., 2015). N = 4 rats. S.E.M. values provided. One-tailed paired *t*-test was performed. A range is provided in the reference values to account for the slow and fast dopamine domains (dorsal striatal measurements based on medial forebrain bundle stimulation).

Parameters	Best-fit estimates (cocaine)		*p*-value
	Pre-drug	Post-drug	
Dopamine release per stimulus pulse, R_P_ (mols X 10^–21^)	6.98 ± 3.26	16.0 ± 3.34	0.093
Modifier for dopamine release, k_R_ (s^−1^)	−0.65 ± 4.72	−0.18 ± 0.20	Comparison cannot be made as some values were zero
Constant for dopamine uptake, k_U_ (s^−1^)	1.08 ± 0.53	0.36 ± 0.09	0.028
Constant for dopamine transport, k_T_ (s^−1^)	1.40 ± 0.22	1.40 ± 0.42	0.428

### Statistical Analysis

Statistical analysis was performed using ratio two-tailed paired *t*-tests (PRISM 8, GraphPad). Significance was set at *p* < 0.05. In the phasic experiments, three planned paired *t*-tests were performed (response at 5 min after cocaine vs baseline, saline, 60 min post-cocaine). In the tonic experiments, the peak level after cocaine injection was compared to baseline and saline levels.

## Results

### Phasic Response and Dopamine Dynamics

**FIGURE 2:**
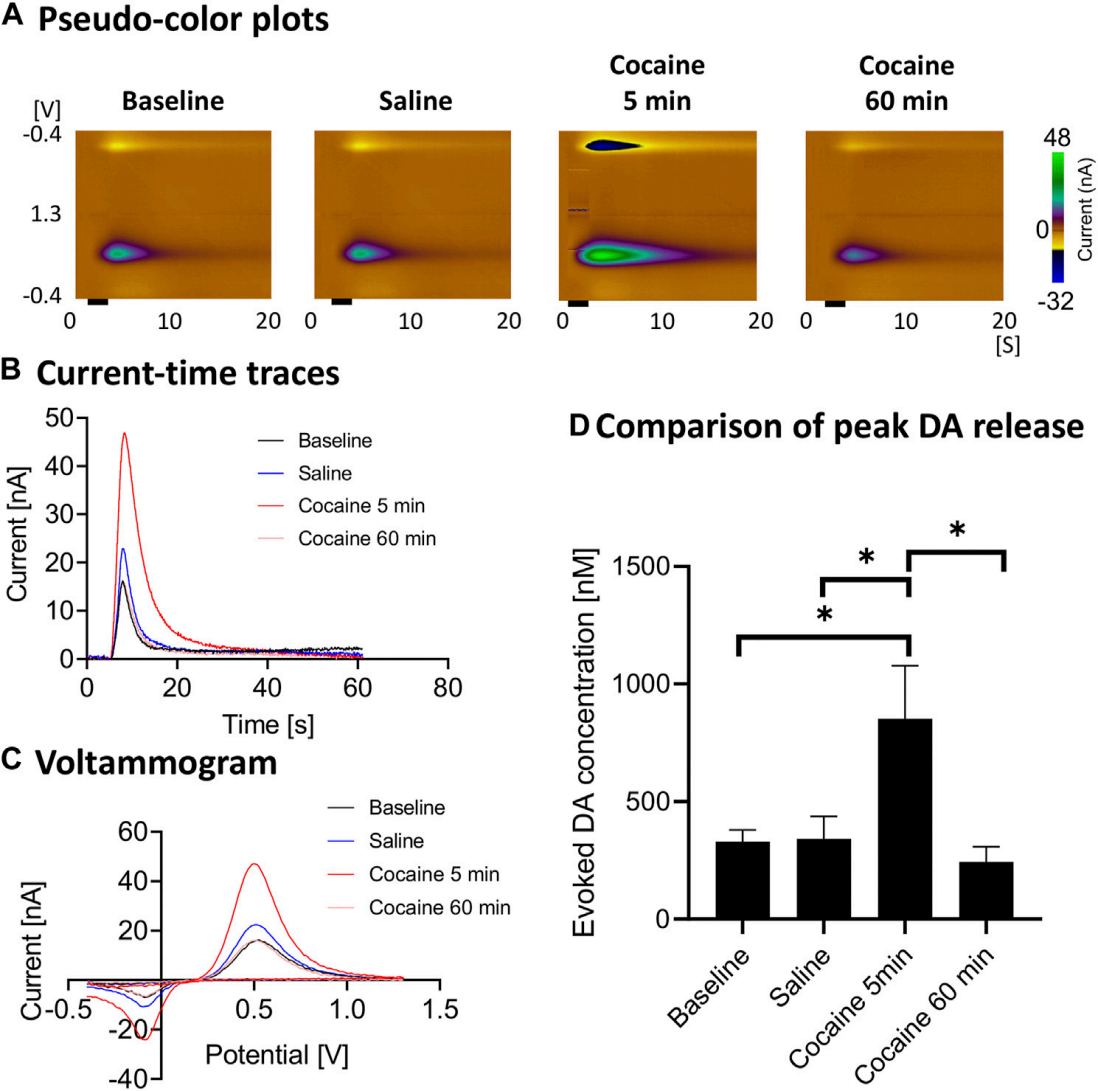
Peak and gradual decay of cocaine-induced changes in stimulation-evoked dopamine release. *In vivo* FSCV measurements in the nucleus accumbens core showing augmented dopamine responses to cocaine injection (3 mg/kg, i.v.). Responses were measured following medial forebrain bundle stimulation (2 s, biphasic, 300 μA, 2 ms pulse width). **(A)** Representative pseudo-color plots pre- and post-cocaine injection, **(B)** Oxidative current-time traces, **(C)** Voltammograms (at the peak) and **(D)** Maximum change in dopamine concentration with medial forebrain bundle stimulation at different time points (*n* = 4 rats). Black bar represents electrical stimulation (2 s). *denotes *p* < 0.05. *n* = 4 rats. *DA*, dopamine.

Cocaine administration consistently led to enhancement of stimulation-evoked dopamine responses (Figure 2). The evoked phasic response at 5 min after cocaine injection was significantly higher than saline control (ratio two-tailed paired *t-*test, *p* = 0.0124, *n* = 4 rats), baseline (ratio two-tailed paired *t-*test, *p* = 0.0326, *n* = 4 rats) and 60 min after cocaine administration (ratio two-tailed paired *t-*test, *p* = 0.0225, *n* = 4 rats). With repeated stimulation performed for 60 min after injection, the peak level dropped to the pre-injection baseline level (Figures 2B,D).

Using the restricted diffusion model discussed in the Methods section, the reuptake parameters for our experiments were calculated both before and after cocaine administration (Table 1). This model includes dopamine release per stimulus pulse and kinetic terms for dopamine reuptake, transport, and release. Cocaine, a dopamine reuptake inhibitor, would be expected to decrease the kinetic parameter for reuptake. Indeed, this is what was found (*p* = 0.028) (Table 1). Cocaine administration did not significantly influence the values of the other kinetic parameters.

### Tonic Response

**FIGURE 3:**
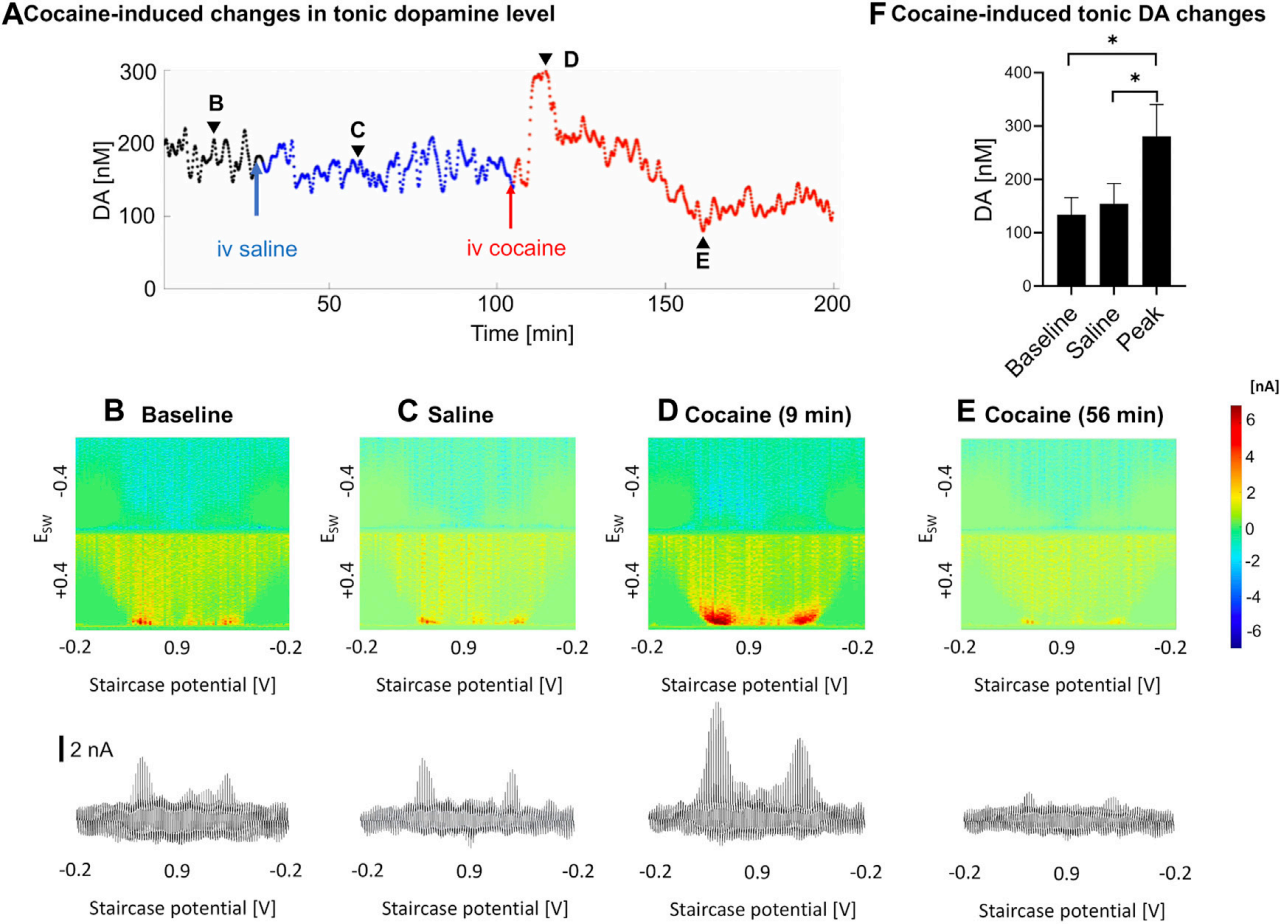
An example of tonic dopamine measurements obtained from the nucleus accumbens core in a single rat with saline then cocaine i.v. injections. **(A)**. Changes in tonic dopamine concentration over time, where the black line denotes the stabilization period, blue line denotes control (saline) and red line denotes post-cocaine measurements. **(B–E)**. High-dimension color plots and voltammograms pre- and post-injections, corresponding to the time points (arrowheads) in **(A)**, respectively, **(F)** Comparison of tonic dopamine concentrations pre- and post-(peak) injection. Ratio two-tailed paired *t-*test, *p* = 0.002, *n* = 5 rats. See supplementary information for a video of the experiment. *DA*, dopamine.

As measured by M-CSWV, a representative example of the temporal changes in tonic dopamine levels in response to cocaine administration is shown in Figure 3. There were variations in the temporal pattern and time to peak changes in concentration (Figure 3A). Baseline recordings were taken for 30 min (Figure 3B), and saline was injected as a control (Figure 3C). Cocaine was injected 90 min later and showed a significant increase in tonic dopamine levels (Figures 3D–F). The tonic dopamine levels were measured for 60–70 min post cocaine injection. Overall, cocaine injections significantly increased dopamine levels in NAcc from 134 ± 32 nM to 281 ± 60 nM (ratio two-tailed paired *t-*test, *p* = 0.002, *n* = 5 rats) (Figure 3F).

## Discussion

This is the first study to characterize changes in tonic dopamine levels in the NAcc in the presence of cocaine in near real-time with an electrochemical technique *in vivo*. By utilizing the M-CSWV technique with its unrivaled temporal resolution and high spatial resolution provided by CFMs, we have demonstrated a more precise picture of how tonic dopamine dynamics change in response to acute cocaine administration.

### Effects of Cocaine on Phasic Dopamine Release

The main findings of our study (see Figures 2A–D) are consistent with the literature which has shown that cocaine (and opioids) leads to increases in dopamine responses in the core of the nucleus accumbens as measured using FSCV (Aragona et al., 2008; Vander Weele et al., 2014). Aside from the robust increase in stimulation-evoked dopamine release after cocaine administration, dopamine release appeared to drop to below pre-cocaine levels (see Figure 2D). However, this did not reach statistical significance with this sample size. This may be due to blockade of dopamine reuptake by cocaine leading to decreased releasable vesicular pool over the course of the cocaine effect, as well as the effect of continuous stimulation. However, the latter is less likely to be the main contributor, given the synapses were provided at least 10 min to recover between stimulations. Further experiments would help to confirm this phenomenon. Also, the reuptake constant, k_U_ was lowered after cocaine administration, which is expected since cocaine is a competitive antagonist of the dopamine transporter and k_U_ is directly proportional to reuptake rate (see Table 1). Cocaine administration would not be expected to influence kinetic parameters for transport between the synapse and the electrode. Consistent with these expectations, these other parameters were not significantly influenced by cocaine administration.

From previous studies, it was found that the NAcc appears to consist of a patchwork of domains that show distinct dopamine kinetics, each demonstrating slow and/or fast evoked responses when the MFB is stimulated (Shu et al., 2013). The dopamine phasic response within the core is also heterogenous in response to cocaine self-administration (Owesson-White et al., 2009). This may explain why there are differences in our values, compared to values published by Walters et al. (2015), as well as the fact that we used different pharmacological agents. The differences may be accounted for by different stimulation parameters, especially the duration of stimulation. However, they do have similar orders of magnitude, which is expected, as both cocaine and nomifensine act by limiting the reuptake of dopamine. As far as we are aware, no studies have used this model to evaluate the evoked response of cocaine, therefore, our results could provide a benchmark for future studies.

### Effects of Cocaine on Tonic Dopamine Levels

In previous microdialysis studies where cocaine was given acutely (Bradberry et al., 1993; Pontieri et al., 1995), intravenous cocaine led to rapid rise in nucleus accumbens tonic dopamine levels, which peaked at 10–20 min (where dopamine was measured at 10–20 min intervals). Peak levels of dopamine varied between 150 to 400% of baseline. In addition, in pharmacokinetic studies, cocaine was eliminated in a biexponential manner after i.v. administration with mean elimination half-lives of 4.4 and 24.8 min, with a rapidly decaying serum concentration (Ma et al., 1999; Sun and Lau, 2001). In our study, a trough was observed in a subset of samples after the peak but not in all (see Figure 3A). This may be due to dopamine depletion after the sharp increase. This may be analogous to the small drop in the mean phasic response at ∼60 min compared to pre-cocaine baseline. Further experiments are needed to confirm this phenomenon.

Previous studies have shown that dopamine release varies widely among test subjects and within dopaminergic structures (Verheij et al., 2008; Owesson-White et al., 2009; Shu et al., 2013), necessitating a trial-and-error approach where the depths of CFM and stimulating electrodes are continually adjusted until the so-called “hotspot” is found. This hotspot is thought to occur when the CFM is close to a site of large synaptic release of dopamine. Variations in the location and behavior of these hotspots may explain the variable effects we see after cocaine administration in our study.

Overall, the present results suggest that M-CSWV can measure drug-induced changes in tonic dopamine levels with high temporal and spatial resolution when compared to microdialysis. Most of these studies used microdialysis with a temporal resolution of 10–20 min (Bradberry et al., 1993; Pontieri et al., 1995; Cadoni et al., 2000). Despite recent developments to reduce the resolution from 20 min down to 1 min (Gu et al., 2015; Ngo et al., 2017), M-CSWV still provides a much higher time resolution with the added benefit of spatial resolution and minimal tissue disruption when used with CFMs.

It is important to note however, that there are two major differences in our M-CSWV results in comparison to microdialysis. First, the tonic dopamine concentrations are very different in magnitude. Our baseline dopamine levels, determined by post-*in vivo* calibration, were at around 100–200 nM; whereas microdialysis studies commonly report values between 10 to 20 nM (Bradberry et al., 1993; Cadoni et al., 2000). Although the order of magnitude differs by a factor of ten, our values are broadly consistent with previous accumbal and striatal dopamine concentrations measured by various electrochemical techniques (Blaha, 1996; Atcherley et al., 2015; Johnson et al., 2017; Oh et al., 2018; Taylor et al., 2019; Barath et al., 2020). The possibility that other interferents such as norepinephrine, which has similar electrochemical properties as dopamine, may be a contributing factor in the differences is unlikely since other studies have demonstrated that the amount of norepinephrine and serotonin in the NAcc is comparatively low (Andrews and Lucki, 2001; McKittrick and Abercrombie, 2007; Zhang et al., 2020). Therefore, the disparities likely represent the fundamental differences between microdialysis and electrochemical techniques. Previous studies have suggested that the traumatized layer of tissue of the order of 200 µm caused by the relatively large diameter of the microdialysis probe may lead to a reduction in dopamine extraction, although relative changes could still be measured (Peters and Michael, 1998; Bungay et al., 2003; Borland et al., 2005). This is minimized by the relatively small diameter of the carbon fibers used in voltammetry. Being able to identify tonic values may help to quantify differences between subjects, as well as the diagnosis of different pathologies, especially given some neuropathological diseases are known to be driven by degeneration and depletion of neurotransmitters such as dopamine (Denys et al., 2004; Beitz, 2014; Oliva and Wanat, 2016; Maia and Conceicao, 2018). With these advantages, there is a strong argument that dopamine levels measured by M-CSWV can serve as important biomarkers for monitoring treatments with rapid bioavailability such as deep brain stimulation.

The other major difference is the relative change in magnitude. Rather than a 400% increase as in the described literature, in our study the dopamine signal mostly doubled. This may again be attributed to the underestimation of baseline in microdialysis, as well as the possibility that cocaine-induced increases in synaptic dopamine may be affected by factors related to the physical presence of the microdialysis probe, such as the formation of a layer of traumatized tissue (Di Chiara et al., 1993; Blaha et al., 1996). The rate and dose of drug administration may also have an impact (Minogianis et al., 2019). As mentioned in the Methods section, the rate was controlled at 5-min duration to avoid overdosing. The use of different anesthetic agents in other studies, such as chloral hydrate, may also lead to discrepancies in results (Bradberry et al., 1993).

Our study focused on the NAcc, so the results should not be generalized to the nucleus accumbens shell, since they are distinct subdivisions of the accumbens or other regions. For example, in one *in vitro* FSCV study, dopamine uptake in the shell was approximately one-third of that measured in the core, and the former was less sensitive to both cocaine and nomifensine (dopamine reuptake inhibitor) (Jones et al., 1996). Also, intravenous cocaine increased extracellular dopamine in the shell more markedly than in the core of the rat nucleus accumbens. Another study utilized immunochemistry to demonstrate that dopaminergic axons in the shell contained lower densities of dopamine transporter than those in the core (Nirenberg et al., 1997). This suggests a more tightly regulated phasic dopamine transmission in this subregion and highlights the value of both phasic and tonic measurements across both regions for future work. In addition, Dreyer *et al.* utilized a computer model to interpret *in vivo* FSCV data from the NAcc and shell after rodents were administered cocaine (Dreyer et al., 2016). After studying the dynamics involved in presynaptic terminal autoreceptor feedback, they concluded that extracellular dopamine concentrations in the core resulted from constant dopamine firing, whereas the shell concentrations reflected dynamic firing patterns. This supported our decision to record from the NAcc using this new tonic dopamine measurement method.

While our technique focuses on a single analyte, dopamine, and therefore may not be as comprehensive as microdialysis studies, precise measurement of dopamine alone is still highly important. Dopamine has a major role in the reward circuit and is one of the key neurotransmitters in the pathophysiology of addiction (Berke and Hyman, 2000). Newer techniques have also been devised to voltammetrically measure serotonin with high sensitivity and selectivity, which can be incorporated in future study designs (Shin et al., 2020). DLight, which is a new technique that uses genetically encoded indicators based on fluorescent proteins with microscopy also allows measurements of neurochemicals with high temporal resolution (Patriarchi et al., 2018). However, the need for a viral vector currently limits its potential use in human subjects.

Another notable characteristic of this study is that the animal experiments were performed under anesthesia in an acute setting using a single dose of cocaine. While we appreciate that addiction is often secondary to chronic drug use in humans, acute experiments offer insight into the first step of the pathophysiological process. Additionally, this study paves the way for future chronic experiments by proving the feasibility of this technique to study the effects of other drugs of abuse. In the future, it is our intention to apply the M-CSWV intraoperatively, particularly in the context of neurological (e.g., Parkinson’s disease) and psychiatric (e.g., addiction) disorders.

Although cocaine is known to enhance dopamine transmission in the nucleus accumbens, this is the first study that utilized M-CSWV to measure accumbal tonic dopamine levels, and to characterize the effect of cocaine on these levels in near real-time. Overall, this technique provides unprecedented insight into the temporal changes in dopamine dynamics, and it will likely be of much value in future addiction studies.

Supplementary video. An example of tonic dopamine measurements obtained from the NAcc in a single rat with saline then cocaine i.v. injections. Upper panel shows the color plot and the lower panel shows the changes in tonic dopamine concentration over time, where the black line denotes the stabilization period, blue line denotes control (saline) and red line denotes post-cocaine measurements.

## Data Availability

The data presented in the study are included in the article, further inquiries can be directed to the corresponding author.
